# Infrared and visible image fusion via octave Gaussian pyramid framework

**DOI:** 10.1038/s41598-020-80189-1

**Published:** 2021-01-13

**Authors:** Lei Yan, Qun Hao, Jie Cao, Rizvi Saad, Kun Li, Zhengang Yan, Zhimin Wu

**Affiliations:** 1 Key Laboratory of Biomimetic Robots and Systems, School of Optics and Photonics, Beijing Institute of Technology, Ministry of Education, Beijing, 100081 China; 2 State Key Laboratory of Precision Measurement Technology and Instruments, Department of Precision Instruments, Tsinghua University, Beijing, 100084 China; 3 School of Mechanical and Electrical Engineering, Shenzhen Polytechnic, Shenzhen, 518055 China

**Keywords:** Imaging and sensing, Mid-infrared photonics

## Abstract

Image fusion integrates information from multiple images (of the same scene) to generate a (more informative) composite image suitable for human and computer vision perception. The method based on multiscale decomposition is one of the commonly fusion methods. In this study, a new fusion framework based on the octave Gaussian pyramid principle is proposed. In comparison with conventional multiscale decomposition, the proposed octave Gaussian pyramid framework retrieves more information by decomposing an image into two scale spaces (octave and interval spaces). Different from traditional multiscale decomposition with one set of detail and base layers, the proposed method decomposes an image into multiple sets of detail and base layers, and it efficiently retains high- and low-frequency information from the original image. The qualitative and quantitative comparison with five existing methods (on publicly available image databases) demonstrate that the proposed method has better visual effects and scores the highest in objective evaluation.

## Introduction

Image fusion is an enhancement technique that aims to combine images obtained from different types of sensor to generate a composite image with substantial information that can be used for human perception or machine vision tasks^1^. Image fusion can be performed at three levels, namely, pixel, feature, and decision levels. In comparison with other approaches, the pixel-level-based image fusion directly combines the original information in the source image to yield more informative fused images for visual perception and computer processing^2^. The pixel-level based method is widely used in pattern recognition^3,4^, remote sensing^5–7^, medical imaging^8,9^, and military applications^10,11^.

Numerous fusion methods have been proposed in the past which achieve good fusion performance. These methods can be classified into four categories based on their adopted theories^2,12^, namely, multiscale transform^13–20^, sparse representation^21–23^, neural network^24–31^, and other optimizations^32–34^. Multiscale transform-based methods decompose source images into several levels, fuse corresponding layers with particular rules, and reconstruct the target images accordingly Popular transforms used for decomposition and reconstruction include wavelet^16^, pyramid^15^ and their derived versions. The multiscale transform-based methods usually fix the decomposition levels, and the adaptive selection of decomposition levels still remains to be solved^12^, For conventional multi-scale transformation, the image decomposition effectively preserves the background information of the image, However, this method lacks effective retention of detailed texture information. Sparse representation-based methods represent images as linear combinations of sparse bases in over complete dictionaries, which is key to their good performance in terms of feature fusion. However, these methods ignore the correlation among different patches, and lead toward the loss of detail information^12^. Neural network-based methods extract image features using artificial neural networks. Artificial neural networks have advantages in image feature processing, and have been applied to image fusion^35–37^. Although deep neural networks efficiently process large-scale object information such as contour, edge, and contrast, they fail to effectively handle the extraction of details, such as textures.

In practice, image fusion has been applied to combine information in infrared (IR) and visible (VIS) images. The potential of VIS images has been limited by poor light and harsh weather conditions (e.g., smog and fog). By contrast, IR images can provide better information in conditions where VIS imaging fails. However, IR images cannot properly reconstruct spatial information about the scene because they operate in a different spectrum that is not visually pleasing. Specifically, the advantage of IR images is their intensity information, which is mainly reflected in the low-frequency information of IR images. The advantages of VIS images are contour and texture information. The contour information is mainly reflected in the low frequency information of the VIS image, and the texture information is mainly reflected in the high frequency information of the VIS image. Thus, the fusion of VIS and IR images at different frequencies can produce complementary information. In addition, through comparative analysis of different methods (presented in the introduction section), we found that the multiscale transform method can realize the decomposition of an image at different frequencies, but the decomposition scale needs to be set manually. Therefore, considering the characteristics of the IR and VIS image fusion, and based on the analysis of four categories of methods, the fusion performance of IR and VIS images can be improved in terms of two aspects: (1) Adaptive decomposition of images by scales; (2) Separation and retention of low-frequency and high-frequency information at different scales.

In this study, an octave pyramid fusion framework is proposed, which achieves two breakthroughs. First, the decomposition levels in the framework realize adaptive selection. Second, interval space decomposition is added in this framework to simultaneously retain lower- and high-frequency information. The proposed fusion framework is a type of multiscale transform that operates in two scale spaces, namely octave and interval spaces. The number of octave spaces represents the level of image decomposition and is adaptive relative to image size. The interval space decomposition performs multiple instances of Gaussian blur on the image to obtain multiple sets of detail and base layers, which retain considerable information about the source image. The proposed framework considers high- and low-frequency information processing for the source image in principle, which effectively improves the performance of the fused image. By doing these, the proposed method effectively improves the quality of image fusion. Experimental results (both qualitative and quantitative) demonstrate the superior fusion performance of the proposed method compared to existing typical schemes.

The remainder of the paper is organized as follows. "Octave Gaussian pyramid" introduces the principle of the octave Gaussian pyramid.  "Image fusion framework based on Octave Gaussian Pyramid" proposes the fusion framework based on the octave Gaussian pyramid. "Experiment and analysis" presents experimental analysis, and compares the performance of our method with five conventional methods over publicly available datasets. Finally, "Conclusion" concludes the paper.

## Octave Gaussian pyramid

The Gaussian function is the only possible scale-space kernel^38^ and it is widely used in image processing. In image fusion, multi-scale transformation based on Gauss decomposition is a classical fusion framework. Generally, Gaussian pyramid is obtained by computing the source images with repeated Gaussian filtering and downsampling. In a traditional Gaussian pyramid, each level is blurred only once by the Gaussian kernel. The entire pyramid contains several detail layers and only one base layer^13^.

The Gaussian blurring of an image is defined as function *L* (*x*, *y*, *σ*_0_), which is generated by convolving variable-scale Gaussian function *G* (*x*, *y*, *σ*_0_) with an input image *I* (*x*, *y*) expressed as:

1 $$ L(x,y,\sigma_{0} ) = G(x,y,\sigma_{0} )*I(x,y), $$

where “ * ” is the convolution operation, *σ*_0_ is the initial blur coefficient, and the Gaussian function is given by:

2 $$ G(x,y,\sigma_{0} ) = \frac{1}{{2\pi \sigma_{0}^{2} }}e^{{ - \frac{{x^{2} + y^{2} }}{{2\sigma_{0}^{2} }}}} . $$

**Figure 1:**
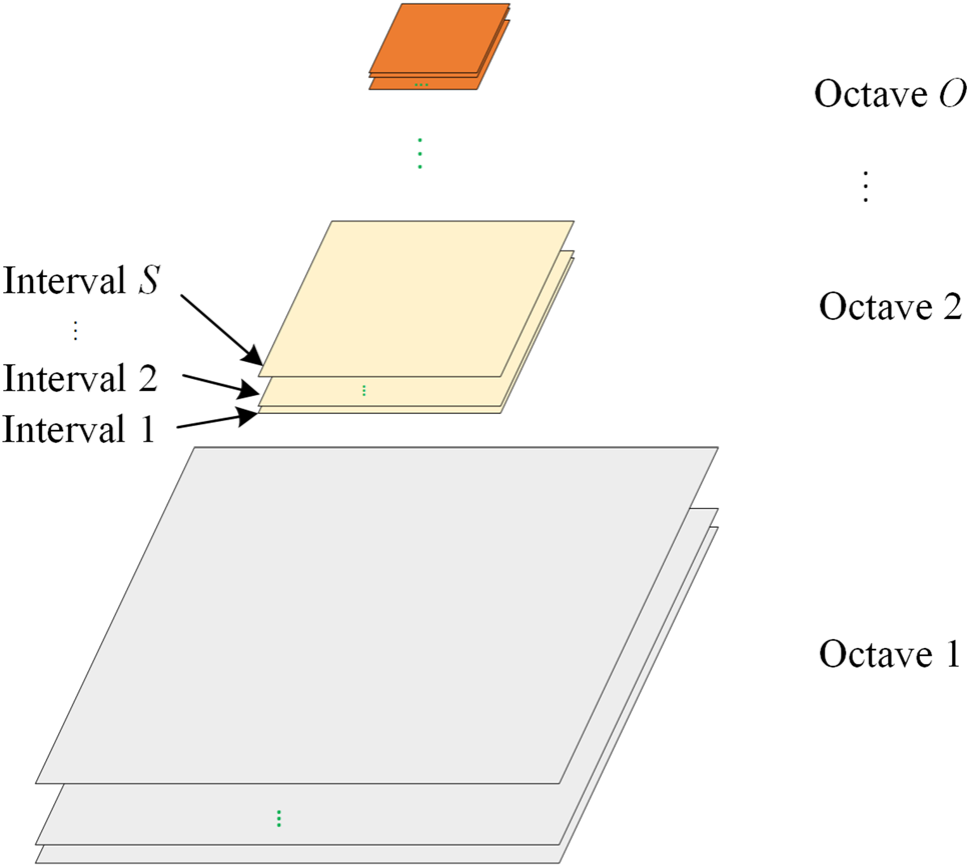
Octave Gaussian pyramid.

The octave pyramid framework proposed in this study contains two scale spaces, namely, octave and interval spaces, as shown in Fig. 1 (where *O* is the number of octaves, and *S* is the number of intervals). In the octave pyramid, two variables are important, namely the number of octaves (*o*) and the number of intervals (*s*) in the octave. The two quantities (*o*, *s*) constitute the scale space of the Gaussian pyramid. Generally, the length and width of an image in the octave are equal. Variable *o* controls the size dimension, whereas *s* distinguishes between images in the same octave scale. The variable *s* also controls different degrees of blur in an octave. Therefore, (*o*, *s*) represents a sample image in the octave Gaussian pyramid.

**Figure 2:**
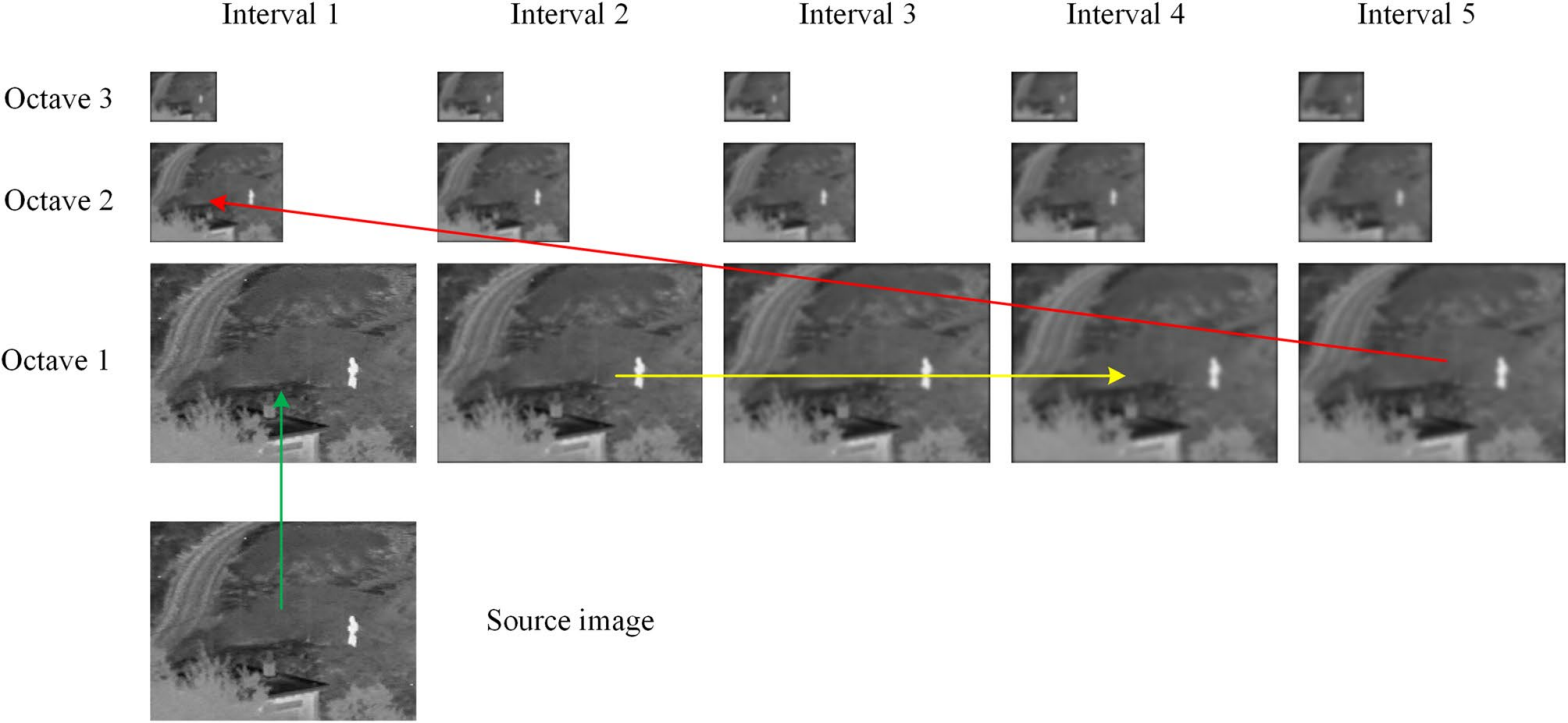
Structure split of Fig. 1

The construction of the octave pyramid is divided into two steps. First, the initial image is blurred with different coefficients to obtain an octave space. Second, the last blurred image in the octave space is downsampled to obtain an image that becomes the initial image in the next octave space (subsequently processed further). The two steps are repeated until the last octave. Figure 2 shows the construction process.

For octave space, the number of octaves is determined adaptively, using the following equation:

3 $$ O = \log_{2} (\min (M,N)) - 2, $$

where *O* is the number of octaves, and (*M*, *N*) is the size of source image. The initial Gaussian blur coefficient of an image in different octaves can be expressed as follows ^38^:

4 $$ \sigma_{o} = 2^{o - 1} \cdot \sigma_{0} {\kern 1pt} {\kern 1pt} {\kern 1pt} {\kern 1pt} {\kern 1pt} {\kern 1pt} {\kern 1pt} {\kern 1pt} {\kern 1pt} {\kern 1pt} {\kern 1pt} {\kern 1pt} {\kern 1pt} {\kern 1pt} {\kern 1pt} {\kern 1pt} o \in [1,O] $$

where “·” is the multiplication operation. Similarly, for the interval space, the Gaussian blur coefficient of each image can be determined by

5 $$ \sigma_{s} = k^{s - 1} \cdot \sigma_{0} ,{\kern 1pt} {\kern 1pt} {\kern 1pt} {\kern 1pt} {\kern 1pt} {\kern 1pt} {\kern 1pt} {\kern 1pt} {\kern 1pt} {\kern 1pt} {\kern 1pt} k = 2^{1/S} {\kern 1pt} {\kern 1pt} {\kern 1pt} {\kern 1pt} {\kern 1pt} {\kern 1pt} and{\kern 1pt} {\kern 1pt} {\kern 1pt} {\kern 1pt} {\kern 1pt} {\kern 1pt} s \in [1,S] $$

where *S* is the number of intervals, and *k* is a constant factor. The Gaussian blur coefficient of an image represented by (*o*, *s*) is:

6 $$ \sigma_{o,s} = 2^{o - 1} \cdot k^{s - 1} \cdot \sigma_{0} . $$

Therefore, the image in the octave Gaussian pyramid can be represented as:

7 $$ L_{o,s} = G_{o,s} *I_{o,1} , $$

and

8 $$ \begin{gathered} I_{o,1} = \left\{ \begin{gathered} I(x,y),{\kern 1pt} {\kern 1pt} {\kern 1pt} {\kern 1pt} {\kern 1pt} {\kern 1pt} {\kern 1pt} {\kern 1pt} {\kern 1pt} {\kern 1pt} {\kern 1pt} {\kern 1pt} {\kern 1pt} {\kern 1pt} {\kern 1pt} {\kern 1pt} {\kern 1pt} {\kern 1pt} {\kern 1pt} {\kern 1pt} {\kern 1pt} {\kern 1pt} {\kern 1pt} {\kern 1pt} {\kern 1pt} {\kern 1pt} {\kern 1pt} {\kern 1pt} {\kern 1pt} {\kern 1pt} {\kern 1pt} {\kern 1pt} {\kern 1pt} o = 1 \hfill \\ down(I_{o - 1,S} ),{\kern 1pt} {\kern 1pt} {\kern 1pt} {\kern 1pt} {\kern 1pt} {\kern 1pt} {\kern 1pt} {\kern 1pt} o \in [2,O] \hfill \\ \end{gathered} \right. \hfill \\ G_{o,s} = \frac{1}{{2\pi \sigma_{o,s}^{2} }}e^{{ - \frac{{x^{2} + y^{2} }}{{2\sigma_{o,s}^{2} }}}} . \hfill \\ \end{gathered} $$

where *down* represents downsampling, and *I*_*o-1,S*_ denotes the final interval of the (*o-*1) octave. Furthermore, the difference-of-Gaussian (DOG) equation can be derived from Eq.(7), as shown as follows:

9 $$ DOG_{o,s} = \left\{ \begin{gathered} L_{{o,s{\kern 1pt} }} {\kern 1pt} {\kern 1pt} {\kern 1pt} {\kern 1pt} {\kern 1pt} {\kern 1pt} {\kern 1pt} {\kern 1pt} {\kern 1pt} {\kern 1pt} {\kern 1pt} {\kern 1pt} {\kern 1pt} {\kern 1pt} {\kern 1pt} {\kern 1pt} {\kern 1pt} {\kern 1pt} {\kern 1pt} {\kern 1pt} {\kern 1pt} {\kern 1pt} {\kern 1pt} {\kern 1pt} {\kern 1pt} {\kern 1pt} {\kern 1pt} {\kern 1pt} {\kern 1pt} {\kern 1pt} {\kern 1pt} {\kern 1pt} {\kern 1pt} {\kern 1pt} {\kern 1pt} {\kern 1pt} {\kern 1pt} {\kern 1pt} {\kern 1pt} {\kern 1pt} {\kern 1pt} {\kern 1pt} {\kern 1pt} {\kern 1pt} s = S \hfill \\ L_{o,s + 1} - L_{o,s} ,{\kern 1pt} {\kern 1pt} {\kern 1pt} {\kern 1pt} {\kern 1pt} {\kern 1pt} {\kern 1pt} s = [1, \cdots ,S - 1] \hfill \\ \end{gathered} \right.. $$

**Figure 3:**
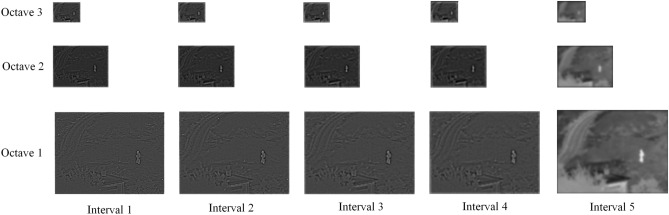
DOG of Fig. 2

Figure 3 shows the DOG image representation for the pyramid in Fig. 2. The image is decomposed into high- and low-frequency information by the octave DOG pyramid. The traditional Gaussian difference pyramid has only one base layer and one group of detail layers. However, in the octave DOG pyramid, the number of base layers is *O*, and (*S*-1) groups of detail layers are present. Specifically, the maximum interval value (e.g., in octave 1 the interval 5 in Fig. 3) in each octave space is the base layer, and the remaining the intervals are detail layers.

## Image fusion framework based on octave Gaussian pyramid

**Figure 4:**
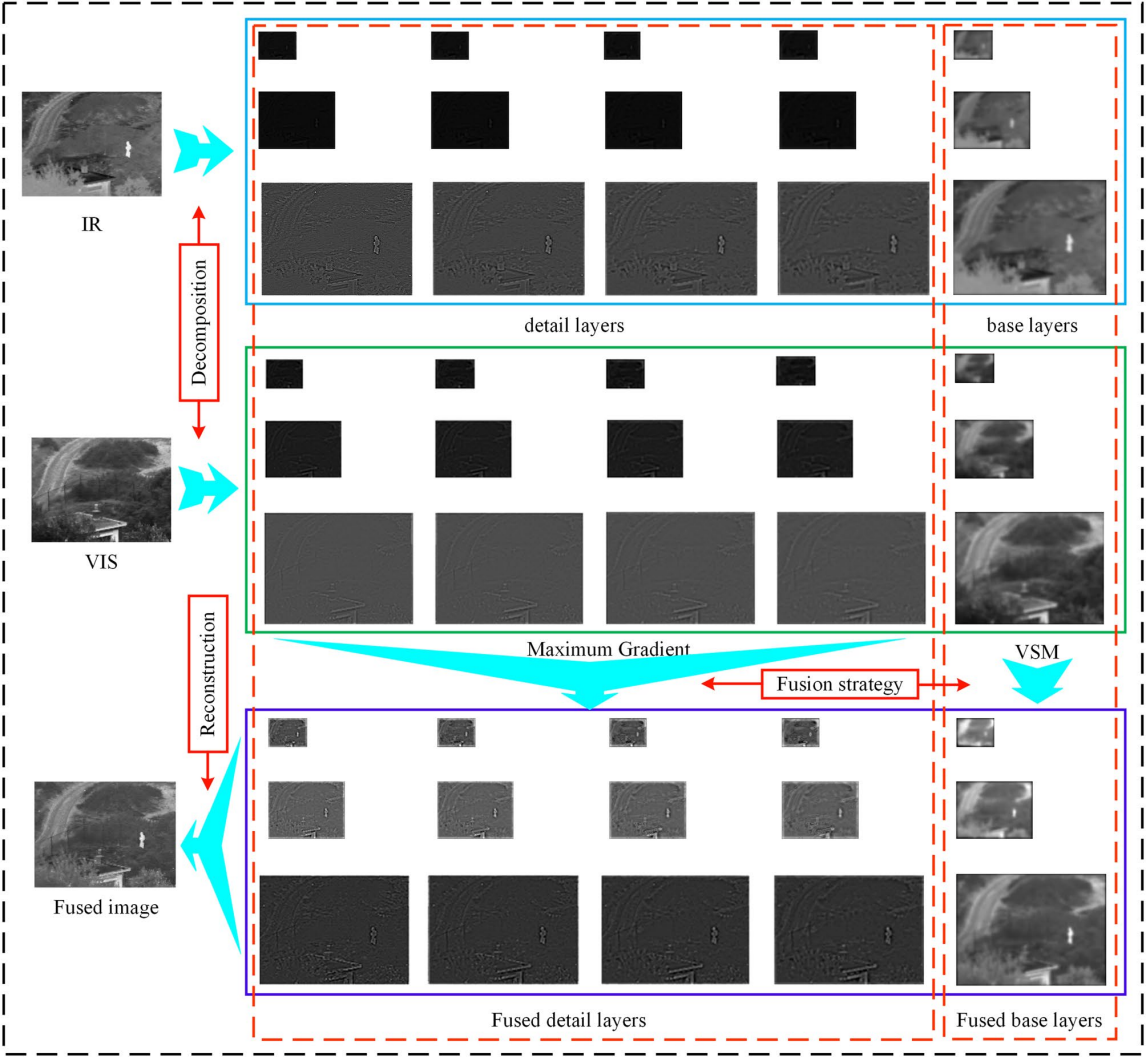
Proposed imaging framework.

Figure 4 summarizes the main stages in the proposed framework. First, using the octave Gaussian pyramid, source images are decomposed into two parts, namely, detail and base layers. Second, the detail layers are fused by the maximum gradient strategy. The base layers use the visual saliency map (VSM) rule for fusion^34^. Finally, the fused image is obtained by reconstructing the fused detail layers and the fused base layers.

### Image decomposition

On the basis of the principle introduced in Sect. 2, the image can be divided into detail layers and base layers using Eq. (9), as shown as follows:

10 $$ \begin{gathered} b_{o,s} = L_{o,s} ,{\kern 1pt} {\kern 1pt} {\kern 1pt} {\kern 1pt} {\kern 1pt} {\kern 1pt} {\kern 1pt} {\kern 1pt} {\kern 1pt} {\kern 1pt} {\kern 1pt} {\kern 1pt} {\kern 1pt} {\kern 1pt} {\kern 1pt} {\kern 1pt} {\kern 1pt} {\kern 1pt} {\kern 1pt} {\kern 1pt} {\kern 1pt} {\kern 1pt} {\kern 1pt} {\kern 1pt} {\kern 1pt} {\kern 1pt} {\kern 1pt} {\kern 1pt} {\kern 1pt} {\kern 1pt} {\kern 1pt} {\kern 1pt} {\kern 1pt} {\kern 1pt} {\kern 1pt} {\kern 1pt} {\kern 1pt} {\kern 1pt} {\kern 1pt} {\kern 1pt} {\kern 1pt} {\kern 1pt} {\kern 1pt} {\kern 1pt} {\kern 1pt} {\kern 1pt} {\kern 1pt} {\kern 1pt} {\kern 1pt} {\kern 1pt} {\kern 1pt} s = S \hfill \\ d_{o,s} = L_{o,s + 1} - L_{o,s} .{\kern 1pt} {\kern 1pt} {\kern 1pt} {\kern 1pt} {\kern 1pt} {\kern 1pt} {\kern 1pt} {\kern 1pt} {\kern 1pt} {\kern 1pt} {\kern 1pt} {\kern 1pt} {\kern 1pt} {\kern 1pt} {\kern 1pt} {\kern 1pt} {\kern 1pt} {\kern 1pt} others \hfill \\ \end{gathered} $$

where *b*_*o,s*_ denotes the base layers and *d*_*o,s*_ represents the detail layers.

For IR and VIS source images, ${b}_{o,s}^{IR}$ *bIR o,s* and *dIR o,s* represent the base layers and detail layers of the IR image, respectively; *bVIS o,s* and *dVIS o,s* represent the base and detail layers of the VIS image, respectively.

### Strategy for image fusion

#### Fusion for base layers

In image decomposition, the base layer contains a wealth of information, such as image texture, contrast, edges, and other background information. The purpose of base layer fusion is to transfer information from the base layer of the IR and VIS images to the fused image. For example, the IR images contain strong contrast information, while the VIS images have rich texture information. The VSM method calculates the importance of each pixel relative to the original image^39^. As a result, the contrast and texture information in the source image can be well preserved and a better base layer fusion effect can be achieved.

VSM defines pixel-level saliency on the basis of a pixel’s contrast to all other pixels. The saliency value *V*^k^(*p*) of pixel p is defined as follows:

11 $$ V^{k} (p) = \sum\limits_{{\forall q \in I^{k} }} {|I_{p}^{k} - I_{q}^{k} |} , $$

where *k* denotes the source images and *k* = {IR, VIS}, *I*_*p*_ denotes the intensity value of pixel *p* in image *I*, and *q* is each pixel of image *I*. The visual saliency of a particular pixel is computed by individually subtracting its intensity value with all the pixels in the image and then summing up those values.

For Eq. (11), the pixel by pixel expansion of *V*^*k*^(*p*) can be written as follows:

12 $$ V(p) = |I_{p} - I_{1} | + |I_{p} - I_{2} | + \cdots + |I_{p} - I_{N} |, $$

where *N* is the number of pixels in *I*. The saliency values are equal if two pixels have the same intensity value, such that Eq. (12) can be rewritten as follows:

13 $$ V^{k} (p) = \sum\limits_{l = 0}^{L - 1} {S_{l} |I_{p}^{k} - I_{l}^{k} |} , $$

where *I* denote pixel intensity, *S*_*l*_ represents the number of pixels whose intensities are equal to *I*, and *L* is the gray levels of images and *L* = 256 in this paper. Furthermore, the visual saliency weight map *V*^*k*^ will be obtained by calculating the visual saliency of other pixels in image using Eq. (13). Finally, the *V*^*k*^ is normalized to [0, 1].

In Eq. (13), we obtain a saliency map for the original image. Regions with large values of VSM typically correspond to intensity and texture areas, whose information are useful and necessary for fusion. The base layer fusion rule is written as

14 $$ F_{{b_{o,s} }} = VSM(b_{o,s}^{{\rm{IR}}} ,b_{o,s}^{{\rm{VIS}}} ) = \frac{{(V_{o,s}^{IR} b_{o,s}^{{\rm{IR}}} + (1 - V_{o,s}^{IR} )b_{o,s}^{{\rm{VIS}}} ) + (V_{o,s}^{VIS} b_{o,s}^{{\rm{VIS}}} + (1 - V_{o,s}^{VIS} )b_{o,s}^{{\rm{IR}}} )}}{2}. $$

where *V*^IR^ and *V*^VIS^ denote the VSM of the IR and VIS images, respectively.

#### Fusion for detail layers

Generally, the method of detail layer fusion uses the maximum pixel value between the corresponding IR and VIS images. However, the details of an image are reflected by its pixel gradient. Therefore, the maximum gradient strategy is used for the detail layer fusion^40^. Specifically, the gradient of image *I* is obtained as follows:

15 $$ \nabla = \sqrt {I_{g}^{2} + I_{{g^{^{\prime}} }}^{2} } $$

where *g* represents the horizontal gradient operator, and *g*' denotes the vertical gradient operator.

For the fusion of detail layers, the pixels with the largest gradient in IR and VIS detail layers are taken as the fusion result. In image processing, ∇*dIR o,s* and ∇*dIR o,s* represent the detail layer gradient in IR and VIS image decomposition, respectively. The fusion strategy can be defined as:

16 $$  F_{{d_{o,s} }} = d_{o,s}^{IR} {\kern 1pt} {\kern 1pt} {\kern 1pt} *{\kern 1pt} {\kern 1pt} {\kern 1pt} (\nabla d_{o,s}^{IR} > \nabla d_{o,s}^{VIS} ) + d_{o,s}^{VIS} {\kern 1pt} {\kern 1pt} {\kern 1pt} *{\kern 1pt} {\kern 1pt} {\kern 1pt} (\nabla d_{o,s}^{VIS} > \nabla d_{o,s}^{IR} ) $$

where “·* ” is the matrix dot multiplication.

### Image reconstruction

Image reconstruction aims to obtain the final fused image. First, the fused base and detail layers are reconstructed into an octave Gaussian pyramid. Then, the first interval in each octave is taken to form a traditional Gaussian pyramid. Finally, the final fused image can be obtained by reconstructing this traditional pyramid.

On the basis of the base and detail layers after fusion, the octave Gaussian pyramid is reconstructed by the following equation:

17 $$ F_{o,s} = \left\{ \begin{gathered} F_{{d_{o,s} }} = F_{{b_{o,s} }} ,{\kern 1pt} {\kern 1pt} {\kern 1pt} {\kern 1pt} {\kern 1pt} {\kern 1pt} {\kern 1pt} {\kern 1pt} {\kern 1pt} {\kern 1pt} {\kern 1pt} {\kern 1pt} {\kern 1pt} s = S \hfill \\ F_{{d_{o,s} }} + F_{{d_{o,s + 1} }} .{\kern 1pt} {\kern 1pt} {\kern 1pt} {\kern 1pt} {\kern 1pt} {\kern 1pt} {\kern 1pt} {\kern 1pt} {\kern 1pt} {\kern 1pt} {\kern 1pt} {\kern 1pt} s = [S - 1,{\kern 1pt} \cdots ,1] \hfill \\ \end{gathered} \right. $$

According to Eq. (7), *L*_*o,s*_ is the decomposition of *L*_*o*,1_. Thus, *L*_*o*,1_ contains all the information in *L*_*o,s*_. Similarly, *F*_*o*,1_ is considered to contain all the information in the pyramid. The fused image will be obtained by:

18 $$ F{ = }\max [F_{{o{ - }1,1}} ,{\kern 1pt} {\kern 1pt} {\kern 1pt} {\kern 1pt} up(F_{o,1} )*G_{{o{ - }1,S}} ].{\kern 1pt} {\kern 1pt} {\kern 1pt} {\kern 1pt} {\kern 1pt} {\kern 1pt} {\kern 1pt} {\kern 1pt} {\kern 1pt} {\kern 1pt} {\kern 1pt} {\kern 1pt} {\kern 1pt} {\kern 1pt} o = [O,O - 1, \cdots ,2] $$

where *up* is the upsampling operation, and “*” represents convolution operation.

## Experiment and analysis

### Experimental setting

**Figure 5:**
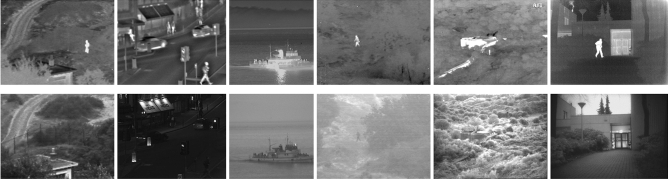
Portion of test images used in our experiments.

To verify the effectiveness of the proposed framework, 21 pairs of IR/VIS images are used in our experiments. Twenty-one pairs of images have been widely used in image fusion research; they are publicly available online^41^. Some sample images from the test set are shown in Fig. 5.

In this study, five typical fusion methods (classified into four categories) are selected for comparison with the proposed fusion framework. These existing methods include dual tree-complex wavelet transform (DTCWT)^14^, ratio of low-pass pyramid (RLP)^13^, convolutional sparse representation (ConvSR)^23^, fusion using deep framework (F_VGG)^27^, fusion by gradient transfer, and total variation minimization (GTF)^32^. The DTCWT and RLP based fusion methods represents the multiscale transform. The ConvSR-based fusion methods use the sparse representation framework, and the F_VGG based fusion approach is a neural network-based method. By contrast, the GTF based fusion uses ‘gradient transfer and total variation minimization’ method, which is different from others.

Objective evaluation plays an important role in image fusion because the performance of a fusion method is mainly assessed by quantitative scores on different metrics. Various fusion metrics have been proposed in recent years. In this study, we quantitatively evaluate the performance of different fusion methods using two quality metrics, namely, multiscale structural similarity (MS_SSIM)^42^, and sum of the correlations of differences (SCD)^43^. The SCD is one of the newly proposed image fusion quality evaluation methods, which calculates quality by considering the source images and their effect on the fused image. The MS_SSIM metric is based on structural similarity, and it provides more flexibility than the single-scale approach does in incorporating the variations of image resolution and viewing conditions. For all metrics, a larger value indicates a better fused result.

### Comparative experiments

#### Influence of octave and interval on fusion

In the proposed framework, multiple decompositions are carried out in the octave and interval space of an image. To explore the influence of the decomposition number of octave and interval on fusion, a comparative experiment was designed and evaluated with SCD metric.

**Figure 6:**
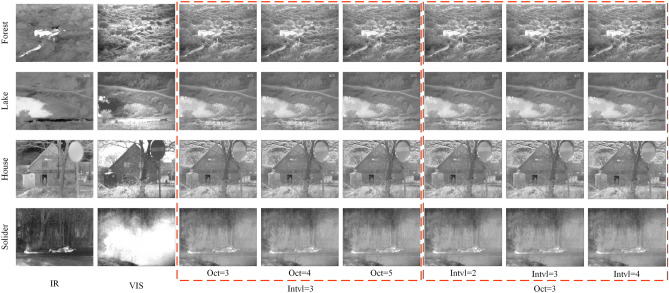
Influence of octave and interval on fusion.

Columns 3 to 5 in Fig. 6 are the experimental results highlighting the influence of octave on fusion. In this experiment, the number of intervals is fixed to 3, and the number of octaves is set to 3, 4, and 5 respectively. The quantitative results are presented in Table 1.

**Table 1:** Influence of octave on fusion.

	Images	Forest	Lake	House	Solider
Interval = 3	Octave = 3	1.6638	1.8155	1.7568	1.7084
	Octave = 4	1.6989	1.8302	1.7768	1.7804
	Octave = 5	1.6996	1.8403	1.7771	1.7814

Columns 6 to 8 in Fig. 6 represents the experiments highlighting the influence of interval on fusion. In this experiment, the number of octaves is set to 3, and the number of intervals is set to 2, 3, and 4 respectively. The quantitative analysis is presented in Table 2.

**Table 2:** Influence of interval on fusion.

	Images	Forest	Lake	House	Solider
Octave = 3	Interval = 2	1.6943	1.8395	1.7757	1.7790
	Interval = 3	1.6976	1.8403	1.7761	1.7802
	Interval = 4	1.6989	1.8415	1.7776	1.7814

In the data analysis of Tables 1 and 2, we find that the fusion effect improves with the increase of octave and interval.

#### Influence of fusion strategy on result

In image fusion, the common fusion strategy for the detail and the base layer are “Average” and “Maximum value” fusion. In the proposed fusion method, the base layer uses the VSM rule, and the detail layer uses the maximum gradient rule. To verify the effectiveness of the fusion strategies adopted in the proposed method, a comparative experiment is designed and evaluated with SCD metric.

**Figure 7:**
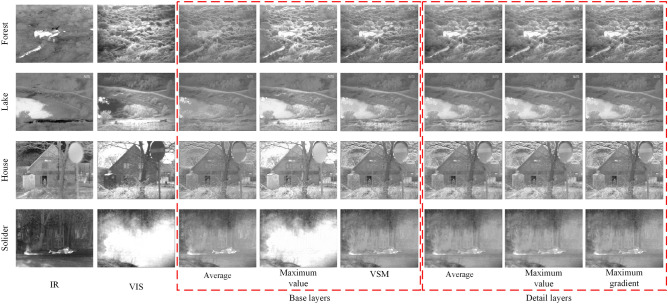
Influence of fusion strategy on result.

Columns 3 to 5 in Fig. 7 are the experimental results obtained by applying different fusion rules (average, maximum values, and VSM) in the base layers. The test results are shown in Table 3. Columns 6 to 8 in Fig. 7 are the experimental results of fusion rules in the detail layer. The test results are shown in Table 4. Analyzing the data in Tables 3 and 4, it can be found that the fusion strategy used in the proposed method performs best. This shows the correctness of the selected fusion strategy.

**Table 3:** Fusion strategies on base layers.

	Strategy	Forest	Lake	House	Solider
Base layers	Average	1.4959	1.6655	1.6947	1.6377
	Maximum value	1.5549	1.6916	1.5305	1.7280
	Maximum gradient	1.6989	1.8402	1.7770	1.7804

**Table 4:** Fusion strategies on detail layers.

	Strategy	Forest	Lake	House	Solider
Detail layers	Average	1.6873	1.8366	1.7748	1.7613
	Maximum value	1.6625	1.8148	1.7555	1.7072
	Maximum gradient	1.6989	1.8402	1.7770	1.7804

### Comparison with other fusion methods

**Figure 8:**
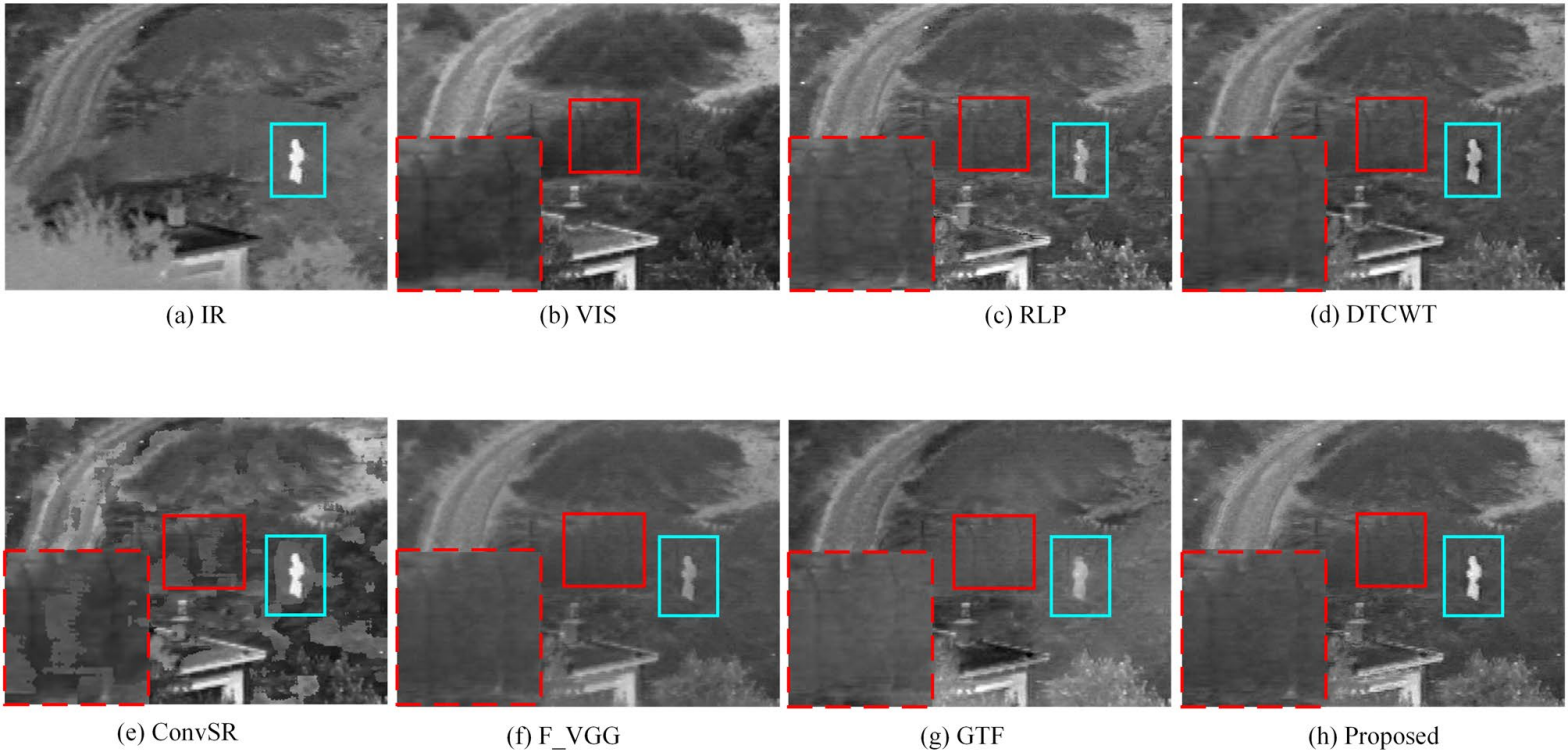
“Sentry” source image pair and their fused images obtained with different fusion methods.

The fused images obtained by the five existing methods and the proposed method are shown in Fig. 8. In the images, the “fence” area is marked with a red border which is enlarged and shown as the image inset in the lower-left corner. The cyan box marks the saliency areas in images.

The images with the highest contrast of the saliency target (cyan frame) are shown in Fig. 8e,h. However, artifacts are found around the saliency target in Fig. 8e. The possible reason is that differences occur between different patches, which leads to artifacts in the reconstruction. Conversely, the contrast of the saliency target in Fig. 8h is similar to that in the IR image. In the other fusion methods, the contrast of the saliency target is lower because the “averaging rule” reduces the contrast of the base layer fusion. Therefore, the fusion strategy based on VSM better retains the contrast of the saliency target in the image.

The “fence” in the red border in Fig. 8 is the textural details of the image; such details are part of high-frequency information. Similarly, artifacts are found in the image in Fig. 8e. In Fig. 8f,g, the “fence” is nearly invisible because the two methods lack effective detail retention capabilities. The “fence” in Fig. 8d,h are clearer than that in Fig. 8c. The “fence” in Fig. 8d,h have the best visual effect. Therefore, the proposed framework has better detail retention capability compared with the other methods.

**Figure 9:**
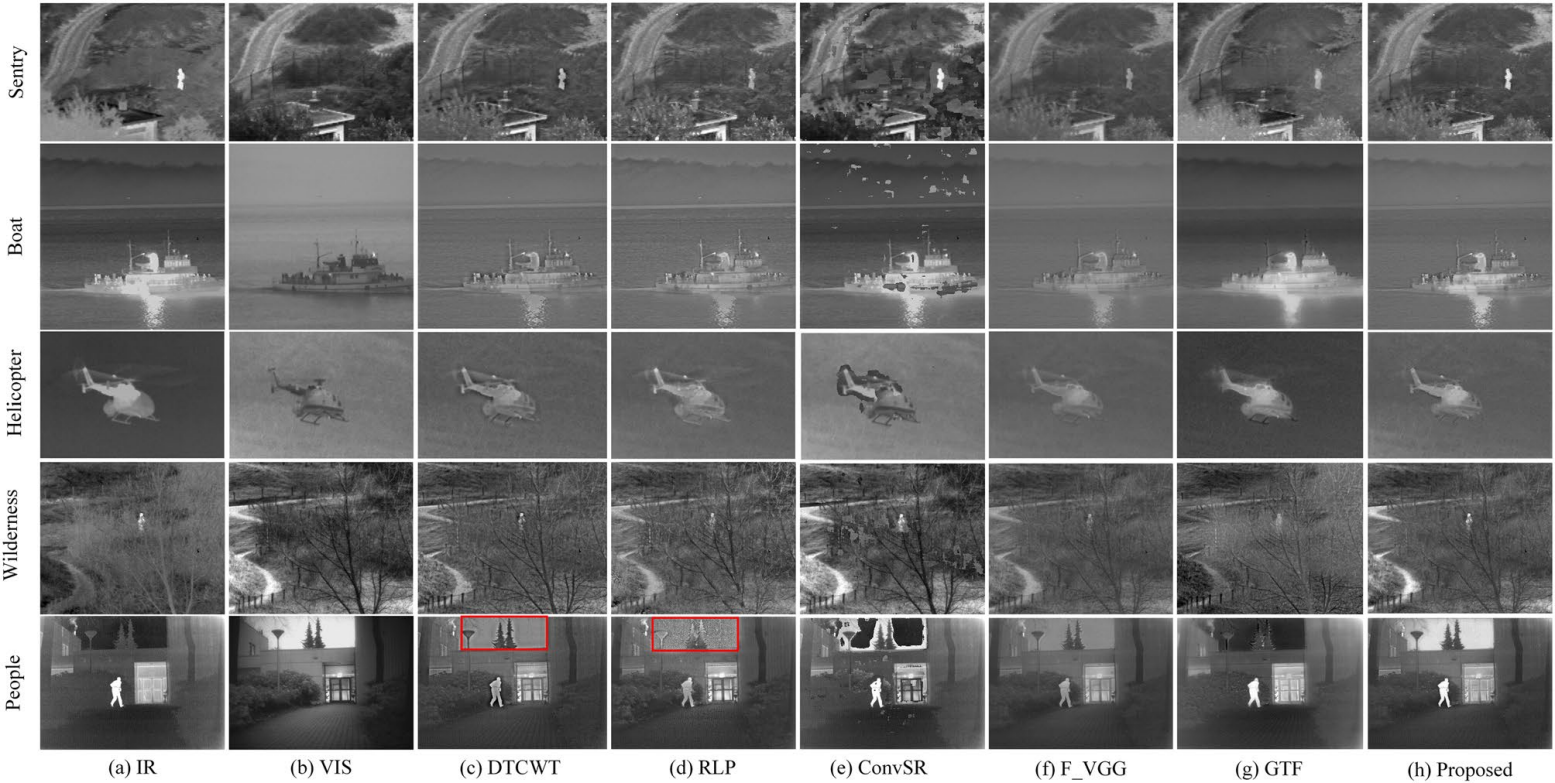
Comparison of fusion results from different methods on the “Sentry”, “Boat”, “Helicopter”, “Wilderness” and “People” source images.

Further comparison of the proposed framework with five other methods is provided in Fig. 9. For each group of results, the first two columns present the original IR and VIS images, respectively, whereas the remaining six columns correspond to the fusion results of the other six methods. As shown in Fig. 9, the results of ConvSR method produce artifacts. In the fusion results shown in Fig. 9f,g, the details of the image are not well preserved. In this respect, the fusion methods of the images in Fig. 9c,d,h have improved detail retention. However, in the “People” image, the methods shown in Fig. 9c,d produce a small number of artifacts (positions marked by red boxes), which reduces the quality of fusion. By contrast, our proposed framework does not introduce artifacts when preserving details. In addition, among all the comparison methods, the thermal radiation information in our results is effectively preserved and the contrast is higher.

Table 5 presents the quantitative comparison of the fusion results in Fig. 9. The best results are highlighted in bold. The results indicate that the proposed method outperforms other methods for most of the fused images. The proposed framework only has lower MS_SSIM values compared with the DTCWT method on ‘‘Helicopter” and ‘‘Wilderness” images.

**Table 5:** Quantitative comparison of different fusion methods.

Images		Metric	DTCWT	RLP	ConvSR	F_VGG	GTF	Proposed
Sentry	SCD		1.4815	1.4463	1.0610	1.4842	0.9697	**1.6326**
	MS_SSIM		0.8796	0.8519	0.6941	0.8699	0.7843	**0.9003**
Boat	SCD		1.9082	1.8903	1.1833	1.9096	1.1407	**1.9526**
	MS_SSIM		0.9369	0.9232	0.8262	0.9126	0.8770	**0.9435**
Helicopter	SCD		1.6658	1.6511	1.1060	1.6845	1.3807	**1.7701**
	MS_SSIM		**0.9423**	0.9156	0.8799	0.9200	0.9324	0.9377
Wilderness	SCD		1.6165	1.6165	1.2408	1.6468	1.1066	**1.7606**
	MS_SSIM		**0.9052**	0.8509	0.8195	0.8484	0.8248	0.8852
People	SCD		1.5558	1.5698	1.0417	1.5409	0.7988	**1.7268**
	MS_SSIM		0.9115	0.8377	0.7846	0.8947	0.7979	**0.9195**

Bold values represent the best fusion effect. The larger the value of the evaluation metric, the better the fusion effect.

**Figure 10:**
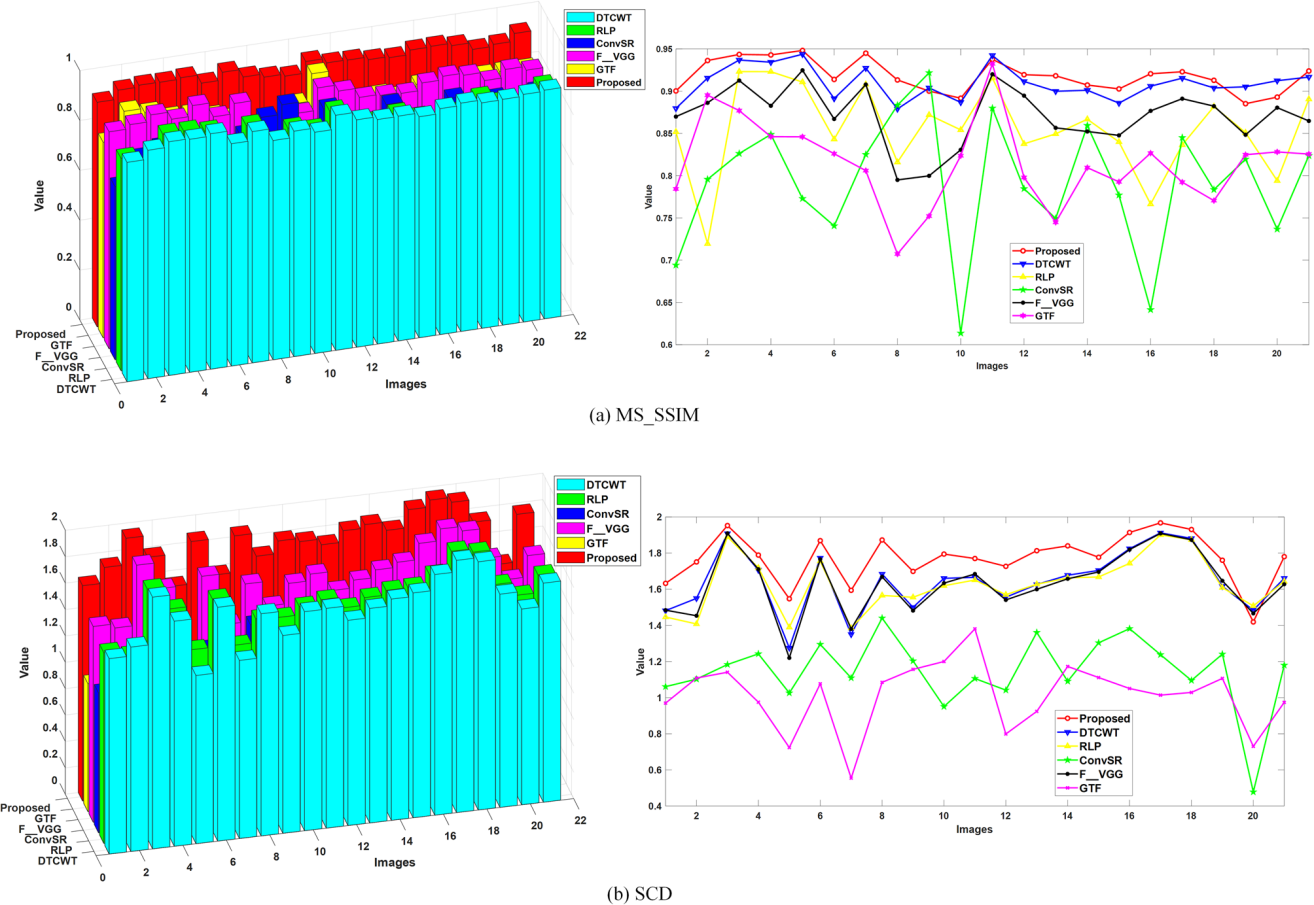
Quantitative comparisons of the metrics (for metrics, larger values indicate better performance).

A fusion comparison test is conducted on all 21 pairs of images. Figure 10a shows the objective comparison of the six existing methods using the MS_SSIM metric. Figure 10b shows the fusion evaluation of 21 pairs of images on the SCD metric. Overall, the proposed method achieves better results in terms of the MS_SSIM metric, followed by the DTCWT method. In terms of SCD, DTCWT and F_VGG methods have similar performance curves, but they remain lower than the proposed framework. Specifically, in the evaluation of the 9th, 11th, 19th and 20th pairs of images in Fig. 10a, our proposed method is slightly lower than that of DTCWT. In the 20th pair of images in Fig. 10b, our method is slightly lower than RLP. In summary, Fig. 10 validates that the proposed method is superior to the other five fusion methods.

## Conclusion

This study presents a fusion framework based on an octave Gaussian pyramid. On the basis of the principle of the octave Gaussian pyramid, the image is decomposed into two scale spaces, namely, octave and interval spaces. Different strategies are used on the decomposed base and detail layers to obtain the fused octave Gaussian pyramid. Finally, the fused image is obtained by restructuring the pyramid. The proposed framework has two obvious advantages: (1) The decomposition level of the image refers to the number of octave spaces in this framework, which realizes adaptive adjustment. (2) Only one set of base and detail layers is used in traditional multiscale decomposition. However, multiple sets of detail and base layers are obtained in the proposed framework. In addition, in this study, the existing fusion methods are divided into four categories. We select typical methods from each category to compare with the proposed framework for comprehensive evaluation. The results testify the effectiveness of our proposed framework.

## Data Availability

The datasets analyzed during the current study are available in https://github.com/hli1221/imagefusion_deeplearning/tree/master/IV_images.
